# Serological and molecular survey of *Leishmania* infection in dogs from Luanda, Angola

**DOI:** 10.1186/1756-3305-7-114

**Published:** 2014-03-24

**Authors:** Hugo Vilhena, Sara Granada, Ana Cristina Oliveira, Henk DFH Schallig, Yaarit Nachum-Biala, Luís Cardoso, Gad Baneth

**Affiliations:** 1 Department of Veterinary Medicine, Escola Universitária Vasco da Gama, Coimbra, Portugal; 2 Animal and Veterinary Research Centre (CECAV), Vila Real, Portugal; 3 Hospital Veterinário do Baixo Vouga, Águeda, Portugal; 4 Clínica Casa dos Animais, Luanda, Angola; 5 Department of Parasitology, Koninklijk Instituut voor de Tropen (KIT)/Royal Tropical Institute, Amsterdam, The Netherlands; 6 Koret School of Veterinary Medicine, The Hebrew University of Jerusalem, Rehovot, Israel; 7 Department of Veterinary Sciences, School of Agrarian and Veterinary Sciences, Universidade de Trás-os-Montes e Alto Douro (UTAD), Vila Real, Portugal; 8 Parasite Disease Group, Instituto de Biologia Molecular e Celular (IBMC), Universidade do Porto, Oporto, Portugal

**Keywords:** Dogs, Luanda, Angola, Direct agglutination test, Polymerase chain reaction, *Leishmania infantum*, Canine leishmaniosis

## Abstract

**Background:**

Canine leishmaniosis (CanL) due to *Leishmania infantum* is a global zoonosis endemic in more than 70 countries in Europe, North Africa, Asia and America; however, data on this infection is scarce from southern Africa. The aim of this study was to survey dogs in Luanda, Angola, for *Leishmania* infection.

**Findings:**

One hundred-and-three dogs presented to a veterinary medical centre in Luanda were serologically and molecularly assessed for *Leishmania* with the direct agglutination test (DAT) and polymerase chain reaction (PCR). Two dogs were seropositive, with DAT titres of 800 and ≥6400; the latter was also found to be PCR-positive and confirmed to be infected with *L. infantum* by DNA sequence analysis. No other dog was found to be PCR-positive. The first dog had been imported from Portugal, but the latter had never left Angola (neither had its parents), strongly suggesting an autochthonous infection.

**Conclusions:**

Although other cases of CanL have previously been described in the country, this is the first reported study of canine *Leishmania* infection at the population level, as well as the first report on the molecular characterization of *L. infantum* in dogs from Angola.

## Findings

The leishmanioses are diseases caused by protozoa of the genus *Leishmania*, which parasitise several mammalian species, including humans. *Leishmania infantum* (syn. *Leishmania chagasi*) is the agent of zoonotic visceral leishmaniosis, in southern Europe, North Africa, the Middle East, Central and South America, Central Asia and China; while *Leishmania donovani* is the agent of anthroponotic visceral leishmaniosis mainly in the Indian subcontinent and East Africa [1].

Canine leishmaniosis (CanL) due to *L. infantum* is a global zoonosis regarded as endemic in more than 70 countries in Europe, Africa, Asia and America, with dogs being the main reservoir for the human infection [2-5]. CanL may also be important in non-endemic countries where introduction of infected or sick dogs constitutes a problem of both veterinary and public health importance [6,7]. Phlebotomine sand fly insects of the genus *Phlebotomus* are the vectors of *Leishmania* spp. in the “Old World”, i.e. Europe, Africa and Asia [8].

**Figure 1:**
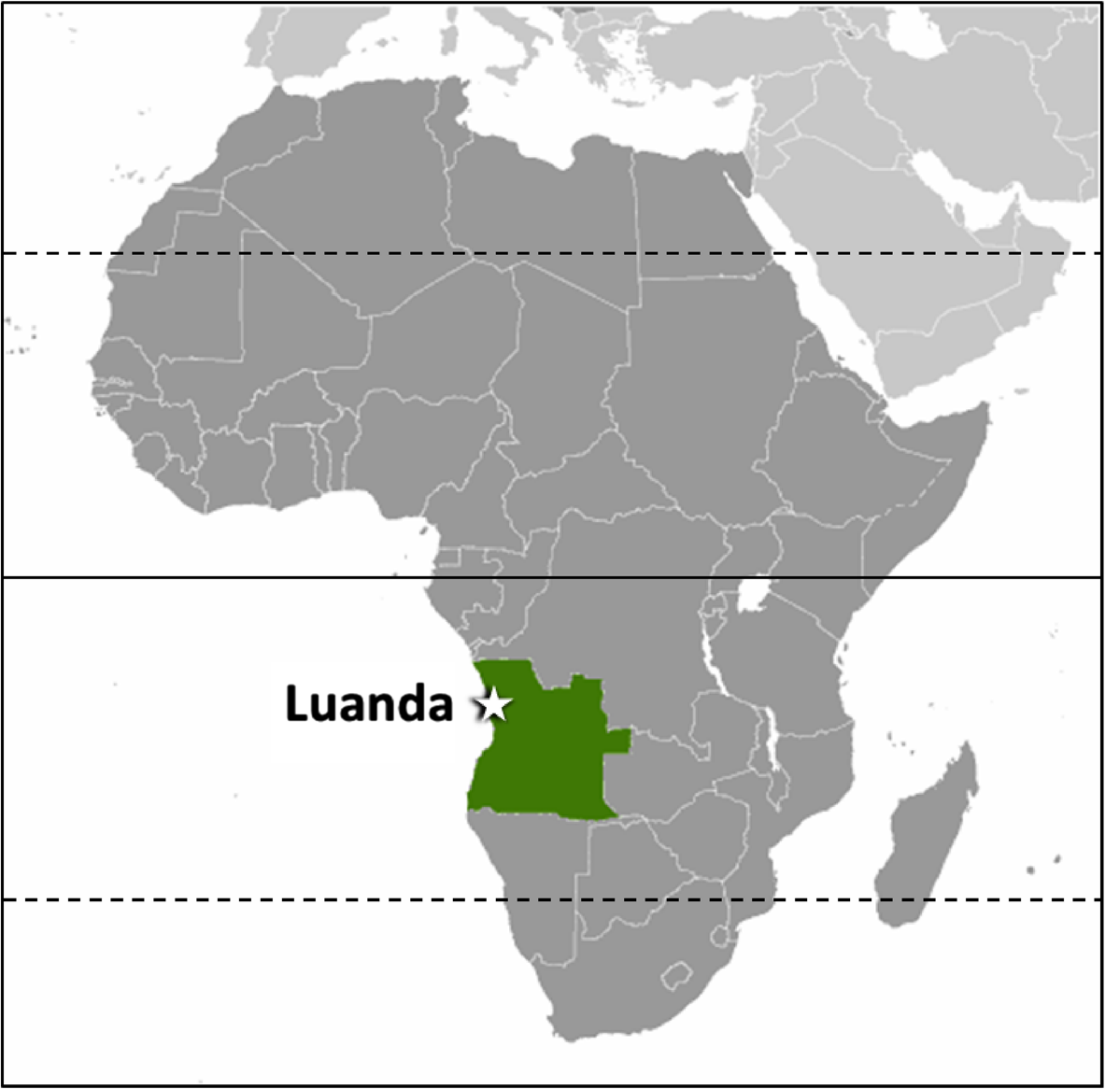
Map of Africa with the location of Angola (green) and the city of Luanda.

Epidemiological surveys in dogs and clinical cases of CanL have been reported in North Africa [9-12], but also in countries of West [13,14] and East Africa [15-18]. Information from Angola (Figure 1) on *Leishmania* spp. infection in dogs and humans is scarce. A small number of human visceral leishmaniosis cases have been described in the scientific literature [19-21] and only five cases of CanL have been reported in dogs from Angola. During the 1960s and 1970s, three of those cases occurred in imported dogs and two others were presumed autochthonous cases [21].

The objective of this study was to perform a serological and molecular survey of *Leishmania* infection in dogs from Luanda, Angola.

One hundred-and-three dogs presented to a veterinary medical centre in the city of Luanda (Figure 1) were assessed. Based on physical examination and clinicopathological data, 50 dogs were classified as apparently healthy and 53 dogs as clinically suspect of a canine vector-borne disease (CVBD). The former were presented for prophylactic procedures, including vaccination and deworming, or for elective surgery; the latter had anorexia, weight loss, fever, dehydration, onychogryphosis, lymphadenomegaly, gastrointestinal alterations, anemia, thrombocytopenia, leukocytosis or leukopenia, hyperproteinemia, hyperglobulinemia, jaundice, and dermatological or ocular disease manifestations. Whenever available, data on gender, age, breed, living conditions and travel history was registered for each dog. The population tested comprised 62 males and 41 females, with ages ranging from 3 months to 14 years (mean ± standard deviation: 2.74 ± 3.21 years). Eighty-eight (85.4%) were pure breed dogs, mainly German Shepherds (n = 32), Rottweilers (n = 12), Boerboels (n = 8), Pit Bulls (n = 7) and Labrador Retrievers (n = 7). Blood samples were collected in EDTA and centrifuged; two thirds of the plasma volume was separated from cells, and both plasma and cells were frozen at -20˚C. Sample collection was carried out in January and February 2013; serology in March 2013; DNA extraction in July 2013; and molecular analysis in September 2013. Owners provided their informed consent for inclusion of their animals in the study, which had been approved by the scientific council of the Vasco da Gama University School as complying with the Portuguese legislation for the protection of animals (Law no. 92/1995).

The direct agglutination test (DAT) for titration of immunoglobulin G antibodies specific to *Leishmania* followed a previously described protocol [22], using a standard freeze-dried antigen at a concentration of 5 × 10^7^ promastigotes per milliliter (KIT Biomedical Research, Amsterdam, The Netherlands). Plasma twofold dilution series ranging from 1:100 to 1:6400 were tested. Results are expressed as an antibody titre, i.e. the reciprocal of the highest dilution at which agglutination (large diffused blue mats) is still clearly visible after 18 h of incubation at room temperature. A cut-off titre of 400 was chosen for seropositivity [22].

DNA was extracted from concentrated blood samples with commercial kits (E.Z.N.A.® Blood DNA Mini Kit, Omega Bio-Tek, Norcross, GA, USA), according to the manufacturer’s instructions. A 265 bp fragment within the internal transcribed spacer 1 (ITS1) region of the *L. infantum* rRNA operon was amplified by real-time polymerase chain reaction (PCR) using primers ITS-219 F (5′-AGC TGG ATC ATT TTC CGA TG-3′) and ITS-219R (5′-ATC GCG ACA CGT TAT GTG AG-3′) and then evaluated by high resolution melt (HRM) analysis [23]. The PCR reaction was performed in a total volume of 20 μl containing 5 μl DNA, 200 nM of each primer, 10 μl Maxima Hot Start PCR Master Mix (2X) (Thermo Scientific, Epsom, Surrey, UK), 50 μM of SYTO9 solution (Invitrogen, Carlsbad, CA, USA) and sterile DNase/RNase-free water (Sigma, St. Loius, MO, USA), using a StepOnePlus real-time PCR thermal cycler (Applied Biosystems, Foster City, CA, USA). Initial denaturation for 5 min at 95°C was followed by 50 cycles of denaturation at 95°C for 5 s, annealing and extension at 59°C for 30 s, and final extension at 76°C for 10 s. Amplicons were subsequently subjected to a HRM step with the temperature being raised to 95°C for 10 s and then lowered to 60°C for 1 min. The temperature was then raised to 95°C at a rate of 0.025°C per second (continuous data acquisition). HRM profiles were analysed using HRM Software (Applied Biosystems). DNA extracted from cell cultures of *L. infantum* and DNA from colony-bred dogs negative by PCR for vector-borne pathogens were used as positive and negative controls, respectively.

PCR products were sequenced using the BigDye Terminator v3.1 Cycle Sequencing Kit and an ABI PRISM 3100 Genetic Analyzer (Applied Biosystems), at the Center for Genomic Technologies, Hebrew University of Jerusalem, Israel. DNA sequences were evaluated with the ChromasPro software version 1.33 and compared for similarity to sequences in GenBank, using the BLAST 2.2.9 program (http://www.ncbi.nlm.nih.gov/BLAST/).

Two dogs were seropositive out of the 103 tested, with DAT titres of 800 (dog 27) and ≥6400 (dog 29), which corresponds to a seroprevalence of 1.9% in the studied population. Dog 29 was also found to be PCR-positive and confirmed to be infected with *L. infantum* by sequence analysis, with a 99% homology with GenBank closest accession number (KC347301.1). No other dog was found to be PCR-positive, leading to a molecular prevalence of 1.0%.

Both dogs 27 and 29 were considered clinically sick, presenting clinical signs compatible with leishmaniosis. Dog 27 was an intact Bullmastiff female aged 6 years that lived exclusively outdoors. On clinical history and physical examination, it was presented for anorexia, weight loss, vomiting, diarrhoea and generalized lymphadenomegaly. Although no external parasites were detected at the time of consultation, a previous infestation with ticks was reported by the owner. Dog 29 was a 4-year old intact mongrel female weighing 29 kg that also lived exclusively outdoors. On clinical history and physical examination, it had anorexia, weight loss, fever, skin lesions, bilateral conjunctivitis and generalized lymphadenomegaly and had a recent episode of tick infestation. Dog 27 had been imported from Portugal as a puppy; while dog 29 had never left Angola.

The prevalence of canine infection in the studied population was apparently low (i.e. 1.0% by PCR and 1.9% by serology), but the studied dogs were only client-owned animals and presumably well cared for. Due to this fact, the prevalence of *Leishmania* infection in the overall population of dogs from Angola might be higher.

Dog 29 was born in Angola, the same as its parents and co-habitants, which suggests an autochthonous infection. Dog 27 was imported from Portugal at approximately 3 months of age, and since then it had never left Angola; therefore, it is not clear whether the infection occurred in Angola or in Portugal, a country where CanL is endemic [24,25]. The finding of an imported dog with CanL in Angola relates to the risks associated with the importation of sick or infected animals, which can contribute to the introduction and propagation of pathogens among the local canine populations [6].

Both dogs were clinically sick at the time of presentation and had clinical signs compatible with leishmaniosis. Although, according to the results of the present study, leishmaniosis is apparently not hyperendemic, it should be considered as a differential diagnosis in dogs with compatible clinical signs in Angola.

Cases of human visceral leishmaniosis have been reported from Angola, some of them with a presumed autochthonous origin [21] and one of those cases typed as being caused by *L. infantum *[20]. In addition, phlebotomine sand flies, i.e. *Phlebotomus* spp. of the *Synphlebotomus* group, were detected in southwestern Angola [21]. Worldwide, dogs are the major reservoir of *L. infantum* for humans, and canine infection with this parasite might also be regarded as a public health problem in Angola.

Further investigation, including a larger number of dogs, canine populations from other cities and provinces of Angola, as well as studies in other vertebrate species, including humans, and in potential vectors of *Leishmania* are needed to better characterize infection and disease in this country.

### Conclusions

Other cases of CanL were described in Angola in the 1960s and 70s, but this is the first population study on canine *Leishmania* infection in the country, as well as the first one to characterize *L. infantum* at the molecular level in dogs. Infection among client-owned dogs is apparently not high, but prevalence in the overall canine population of Angola and specific populations might be higher.

## Competing interests

The authors declare that they have no competing interests.

## Authors’ contributions

HV supervised the study, analysed data and wrote the manuscript; SG collected samples, performed DAT and assisted in drafting the manuscript; ACO co-supervised the study, performed clinical examination and collected samples; HDFHS provided DAT antigen and analysed data; YNB performed PCR and sequencing, and analysed data; LC performed DAT, analysed data and revised the manuscript; GB performed PCR and sequencing, analysed data and revised the manuscript. All authors read and approved the final version of the manuscript.
